# Assessment of outer retinal thickness and function in mice after experimental optic nerve trauma

**DOI:** 10.1186/s12886-022-02737-9

**Published:** 2022-12-20

**Authors:** Karin Rose Lypka, Tal Carmy-Bennun, Kimberly N. Garces, Alexander W. Venanzi, Abigail S. Hackam

**Affiliations:** Bascom Palmer Eye Institute, University of Miami Miller School of Medicine, 1638 NW 10Th Ave., McKnight Building Rm 404, Miami, FL 33136 USA

**Keywords:** Retinal ganglion cells (RGCs), Optic nerve crush, Photoreceptors, Electroretinogram, Axon degeneration

## Abstract

**Background:**

Optic nerve trauma caused by crush injury is frequently used for investigating experimental treatments that protect retinal ganglion cells (RGCs) and induce axonal regrowth. Retaining outer retinal light responses is essential for therapeutic rescue of RGCs after injury. However, whether optic nerve crush also damages the structure or function of photoreceptors has not been systematically investigated. In this study, we investigated whether outer retinal thickness and visual function are altered by optic nerve crush in the mouse.

**Methods:**

Wildtype mice underwent optic nerve crush and intravitreal injection of a control solution in one eye with the fellow eye remaining uninjured. Two weeks after injury, the thickness of the ganglion cell region (GCL to IPL) and photoreceptor layer (bottom of the OPL to top of the RPE) were measured using OCT. Retinal function was assessed using flash ERGs. Immunodetection of RGCs was performed on retinal cryosections and RGCs and ONL nuclei rows were counted. Multiple comparison analyses were conducted using Analysis of Variance (ANOVA) with Tukey’s post hoc test and *P* values less than 0.05 were considered statistically significant.

**Results:**

Optic nerve crush injury induced RGC death as expected, demonstrated by thinning of the ganglion cell region and RGC loss. In contrast, outer retinal thickness, photopic and scotopic a-wave and b-wave amplitudes and photoreceptor nuclei counts, were equivalent between injured and uninjured eyes.

**Conclusions:**

Secondary degeneration of the outer retina was not detected after optic nerve injury in the presence of significant RGC death, suggesting that the retina has the capacity to compartmentalize damage. These findings also indicate that experimental treatments to preserve the GCL and rescue vision using this optic nerve injury model would not require additional strategies to preserve the ONL.

## Background

Dysfunction and death of retinal ganglion cells (RGCs) underly vision loss in many blinding diseases, including glaucoma and optic nerve atrophies. A major focus of preclinical studies is developing therapies for experimental injuries to RGCs and the optic nerve. A commonly used injury model in rodent models is optic nerve crush, which induces axonal degeneration followed by Wallerian RGC death, with ~ 50% RGC loss 7 days after injury and > 80% RGC loss by 14 days [1, 2]. The optic nerve crush model has been used to investigate mechanisms of RGC death at the genomic, molecular and cellular levels and to test experimental therapies for promoting RGC neuroprotection and axon regeneration. For example, optic nerve crush in rodents was used to characterize properties of Wallerian degeneration, test cellular reprogramming factors and identify gene expression dynamics during axonal regeneration and acute optic nerve injury [3]. Molecules and signaling pathways have been identified that induce RGC protection and axonal regrowth after optic nerve crush injury, such as Wnt ligands, CNTF and CCL5 [4–7]. In order to fully rescue vision after RGC injury and axonal damage it is vital that the entire retinal circuitry is preserved. Therefore, an important question for developing RGC neuroprotective treatments is whether optic nerve crush injury and subsequent RGC death are also detrimental to the structure and function of the outer retina. Although tissue remodeling has been documented in the retina after optic nerve injury [8], no previous studies have systematically assessed the function or structure of photoreceptors and outer retina after optic nerve traumatic injury. Therefore, it is currently unknown to what extent injury to the optic nerve and GCL in the optic nerve crush model affects the normal function of photoreceptors and inner retinal neurons.

Previous studies on the relationship between outer retina function and RGC disease or optic nerve injury reported variable outcomes. Wang et al. measured retinal layers using frequency-domain optical coherence tomography in patients with glaucomatous damage and found no changes in the inner nuclear layer and photoreceptor layer [9]. Similarly, Colotto et al. analyzed photoreceptor and inner retina electroretinogram (ERG) responses and showed normal a- and b-wave amplitudes in patients with ocular hypertension or open angle glaucoma with moderate visual field loss [10]. Furthermore, flash ERGs were normal in affected eyes of individuals with optic nerve lesions [11, 12]. In contrast, Velten et al. showed that rod photoreceptor a-waves were slightly lower in advanced glaucoma at high flash intensities compared with normal eyes although dark-adapted b-wave amplitudes were normal [13, 14]. Also, in an experimental nonhuman primate glaucoma model, there was minor yet significant thickening of the outer retina [15], but whether increased thickness was associated with decreased function was not demonstrated. Another study using induced glaucoma in the macaque demonstrated mild but not significant reductions in full field a- and b-wave amplitudes, indicating minimal detrimental effects on outer retina function [16]. Additionally, scotopic ERG a- and b-wave amplitudes were equivalent between induced glaucomatous and control eyes in the macaque despite extensive RGC loss and optic nerve atrophy [17].

In animal models of optic nerve injury, comparisons between normal eyes and eyes after optic nerve transection showed similar flash ERG responses in the rabbit [18], and mildly affected multifocal and full field flash ERG responses in the cat [19]. In contrast, photoreceptor a-waves were higher and b-waves were lower in the rat optic nerve transection model [20], and a- and b-waves were both lower in a later study in the rat [21], indicating the effect on the outer retina varies across species. Furthermore, in a rat model in which optic nerves were injured and the animals were subsequently exposed to damaging light, the outer retina had less photoreceptor death than the uninjured eye, suggesting a protective or preconditioning effect after optic nerve injury [22]. Finally, a study comparing three different RGC injuries in rats showed that outer nuclear layer (ONL) thickness was reduced after NMDA-induced excitotoxicity and episcleral vein cauterization but was not affected in the microbead anterior chamber model [23], indicating ONL responses are dependent on the type of GCL injury. To our knowledge, no previous studies have analyzed the structure and function of the outer retina in the mouse in the commonly used optic nerve crush injury model.

The purpose of this study was to investigate whether optic nerve injury and RGC loss leads to secondary changes in the integrity and light responsiveness of the outer retina. Using a combination of OCT, histology and flash ERG, we demonstrated that the structure and function of the outer retina remains normal two weeks after optic nerve injury, despite significant damage to the optic nerve and retinal ganglion cell layer and reduced RGC function. These findings indicate that tissue responses from optic nerve injury do not spread beyond the GCL to the outer retina despite the small distance that separates retinal layers and excessive neuronal death, tissue remodeling and inflammation in the GCL. Therefore, our results demonstrate that incidental effects on photoreceptors are not evident in this injury model, indicating that experimental therapies that protect the GCL benefit from functional outer retinas and would not need to take into account secondary outer retina damage. Furthermore, this work provides new evidence for the capacity of the retina to restrict or adapt to regional injury thereby preventing damage responses from impacting the photoreceptor layer.

## Methods

### Optic nerve crush (ONC) injury

All mouse procedures were approved by the Animal Care and Use Committee at the University of Miami and complied with the Association for Research in Vision and Ophthalmology statement for use of animals in ophthalmic and vision research. Wildtype mice (C57Bl/6 hybrid strain) [6] were housed in a 12 h light:12 h dark environment with free access to food and water. All mice were genotyped to confirm absence of the retinal degeneration *Pde6b-rd1* allele.

Male and female mice at age 7–8 weeks were randomly assigned to the treatment groups. Crush injury to the optic nerve was performed as described in [6, 7], as follows. Mice were anesthetized using isoflurane and after confirming full anesthesia, one drop of 0.5% proparacaine hydrochloride was added to the left eye and a small incision was made in the conjunctiva at the superior posterior region. The tissue was gently moved to expose the optic nerve and then the nerve was crushed ~ 1 mm behind the globe for 5 s with extra-fine self-closing forceps. To simulate a typical experimental treatment procedure in which neuroprotective or control molecules are intravitreally injected [6, 24], the ONC eyes were intravitreally injected with 2 µM of a non-neuroprotective control peptide diluted in sterile PBS in a volume of 2 µl. This peptide corresponds to a protein domain that confers cellular permeability (amino acid code: DRQIKIWFQNRRMKWKKR, Novus Biologicals, Centennial, Colorado) and is frequently used as a control for peptide blockers of innate immunity [25, 26]. Our previous study demonstrated that this control peptide did not exacerbate retinal damage or alter inflammation [25] and it is used here as a control for neuroprotective peptides instead of saline. Erythromycin ointment was topically applied to the treated eye and mice were injected subcutaneously with buprenorphine SR (0.5 mg/mL). Mice with excessive bleeding or ocular swelling at any point after the procedure were excluded from the study. A naive group was anesthetized only without ONC or intravitreal injection.

### Optical Coherence Tomography (OCT) measurement of retinal layer thickness

Retinal layers were measured using spectral domain optical coherence tomography (SD-OCT) (Bioptigen, Research Triangle Park, NC) 2 weeks after optic nerve injury, as described [27]. After anesthetizing the mice, the pupils were dilated and kept moist using saline. Imaging scans of the retina were performed covering a volume of 1.3 × 1.3 × 1.56 mm^3^ centered on the optic disk to generate 100 cross-sectional B-scans. The average outer retina thickness was measured by manually segmenting the OCT images in 70–80 cross-sectional B-scans across the retina from the bottom of the outer plexiform layer (OPL) to the top of the retinal pigment epithelium (RPE) layer using MATLAB software and analytical programs developed by the Ophthalmic Biophysics Center at the University of Miami. Similarly, the average ganglion cell complex (GCC) region was measured from the nerve fiber layer to the inner plexiform layer (IPL) inclusive using manual segmentation of 75 B-scan images spanning approximately 0.5–0.6 mm from the optic disc. Segmentation was performed by investigators who were masked to the identity of the treatment.

### Retinal activity

Outer retinal function was assessed by flash ERG two weeks after optic nerve injury. The UTAS system and EM for Windows software (LC Technologies, Gaithersburg, MD) was used to measure scotopic and photopic responses with a Ganzfield light-emitting chamber, as described previously [28]. The mice were anesthetized with a ketamine/xylazine mixture and pupils were dilated with 2.5% phenylephrine hydrochloride. A reference electrode was inserted subcutaneously in the center of the forehead, a ground electrode was inserted at the base of the tail and corneal silver wire electrodes were placed on both eyes. The mice were placed on a heating pad that was heated by circulating water and body temperature was maintained using a Physitemp controller. Rod photoreceptor-driven responses were recorded in dark-adapted mice exposed to white light flashes ranging from -1 to + 1 log cd s/m^2^ intensity, and cone-driven photopic responses were recorded in light-adapted mice using flashes of green light -1 to + 1 log cd s/m^2^. The responses to 10 flashes of light with an interstimulus time of 5 s were recorded for each intensity and averaged. The a-wave and b-wave amplitudes for each eye were derived from the ERG waveforms.

### Immunohistochemistry

The mice were humanely euthanized by CO_2_ inhalation followed by cervical dislocation and both eyes were removed and incubated in fresh 4% paraformaldehyde for 1 h followed by incubation in 5% sucrose overnight then 1 h each in 10% sucrose then 20% sucrose. The eyes were embedded in Optimal cutting temperature compound (Sakura, Tissue-Tek) in cryomolds and flash frozen. Eyes were cryosectioned into ten micron sections, mounted onto glass slides, and sections from the central region of the eye were immunostained to detect the RGC marker protein RBPMS. After removing the embedding compound by washing in PBS, the slides were incubated in blocking solution composed of 10% goat serum in 0.3% Triton X-100/PBS for 30 min at room temperature. The slides were then incubated in primary antibody (anti-RBPMS antibody, 1 µg/ml, PhosphoSolutions, Aurora, CO, RRID:AB_2492226) diluted in 2% goat serum in 0.3% Triton X-100/PBS, for 16 h in a humidified chamber at 4 °C followed by washing in PBS and incubation with secondary antibody (Invitrogen, 1:600 dilution) for 30 min at room temperature. The slides were washed again then counterstained with 4′6-diamidino-2-phenylindole (DAPI) and covered with a glass coverslip. The retina sections were imaged with a Zeiss fluorescent microscope using equivalent exposure times among experimental groups and negative control (no primary) slides. For RGC counts, RBPMS-positive cells that were also DAPI-positive were counted in the entire retina section in nine sections per each animal and three animals per treatment group. For ONL nuclei row counts, ~ 35 columns of DAPI-stained nuclei were counted in nine sections per animal from the mid-peripheral region of the eye. Investigators were masked to the identity of the treatment during immunstaining, imaging and counting.

### Statistical analysis

Left and right eyes from each animal were considered as individual data points and compared separately. Statistical analyses were performed using GraphPad Prism (GraphPad Software, Inc, La Jolla, CA). Multiple comparison analyses were conducted using Analysis of Variance (ANOVA) with Tukey’s post hoc test and *p* values less than 0.05 were considered statistically significant. Student’s t-test was performed for experiments containing two groups. For analysis within an experimental group, paired comparisons were made between left and right eyes of the same animal and unpaired comparisons were made using average values for left eyes and average values for right eyes. For analysis between experimental groups, unpaired comparisons were made using average values for left eyes and average values for right eyes. Sample sizes are listed in the figure legends.

## Results

The optic nerve crush injury model leads to rapid RGC degeneration and is associated with corresponding reductions in RGC function and GCL thickness [1, 3]. The objective of this study was to determine whether damage to the optic nerve and GCL has detrimental effects on the outer retina. Therefore, we analyzed the structure and function of the outer retina of eyes subjected to optic nerve crush and intravitreal injection compared to fellow eyes that were not injured.

**Fig. 1:**
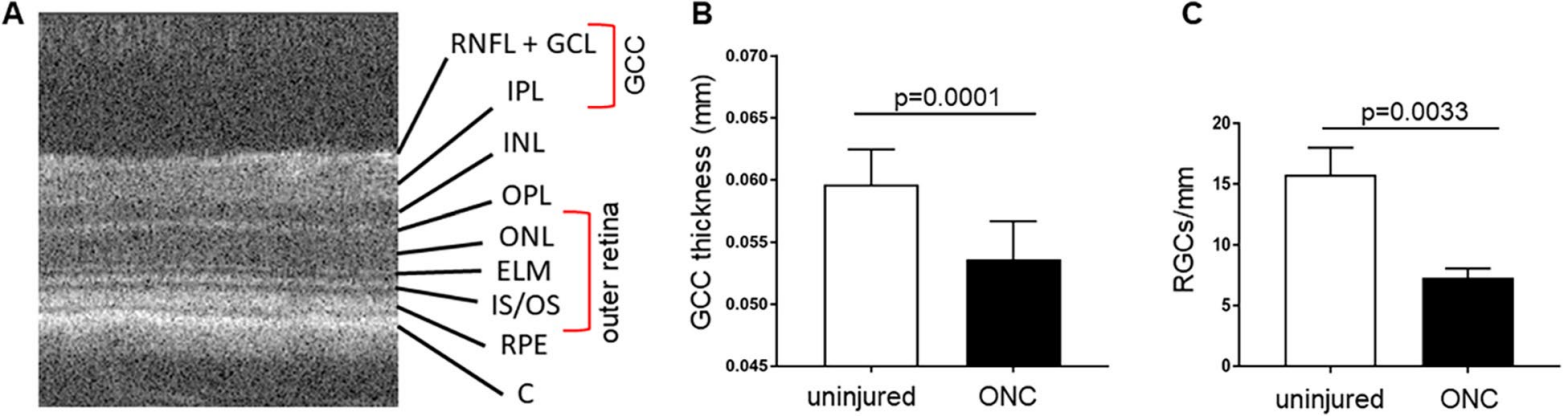
Optic nerve crush injury leads to reduced GCC thickness and lower RGC function. **A** Schematic OCT B scan of a mouse retina depicting the retinal layers. GCC thickness was measured from the nerve fiber layer, ganglion cell layer and inner plexiform layer (NFL/GCL/IPL region). The outer retina thickness was assessed from the bottom of the OPL to the top of the RPE (marked by a red bracket). RNFL, retinal nerve fiber layer; GCL, ganglion cell layer; IPL, inner plexiform layer; INL, inner nuclear layer; OPL, outer plexiform layer; ONL, outer nuclear layer; ELM, external limiting membrane; IS/OS, inner and outer segment of the photoreceptors; RPE, retinal pigment epithelium; C, choroid. **B** OCT imaging was used to measure changes in the thickness of the GCC after optic nerve crush injury compared to fellow eyes. Optic nerve crush (ONC) injury significantly reduced average GCC thickness of injured eyes compared to uninjured eyes. *n* = 7, *p* = 0.0025. **C** RBPMS-positive/DAPI-positive RGCs were quantified in retinal cross-sections of the injured (black column) and uninjured (white column) eyes. Representative images shown in Fig. 4. The number of RGCs/mm were significantly lower in eyes with optic nerve crush as expected (*p* = 0.0033, *n* = 3, unpaired Student’s t-test), consistent with the OCT imaging

To confirm that optic nerve injury led to thinning of the GCL, we used OCT imaging to measure the thickness of the ganglion cell complex (GCC), which is defined as the region extending from the nerve fiber layer to the inner plexiform layer (NFL/GCL/IPL), illustrated in Fig. 1A. Previous studies demonstrated that GCC thickness decreases with RGC death after optic nerve injury and other RGC degenerations [6, 29]. Furthermore, the thickness of GCC region imaged by SD-OCT correlates with cell numbers in the GCL after ONC [30] and optic nerve transection [31]. As shown in Fig. 1B, the average thickness of the GCC in uninjured eyes was 0.05964 ± 0.0028 mm, whereas average GCC thickness in injured eyes was reduced to 0.05361 ± 0.0031 mm 2 weeks after injection (*p* = 0.0025, *n* = 7, unpaired Student’s t-test). Therefore, optic nerve crush caused degeneration of the ganglion cell region as expected. To confirm the loss of RGCs after ONC, we quantified RGCs in injured and uninjured eyes using the RGC-specific marker protein RBPMS (see Fig. 4 for representative images). Retinas after ONC had an average of 7.28 ± 0.45 RGC/mm whereas the uninjured retinas had an average of 15.78 ± 1.28 RGC/mm (*p* = 0.0033, *n* = 3, unpaired Student’s t-test) (Fig. 1C). Therefore, there was a significant reduction in RGCs in eyes with optic nerve crush, as expected.

**Fig. 2:**
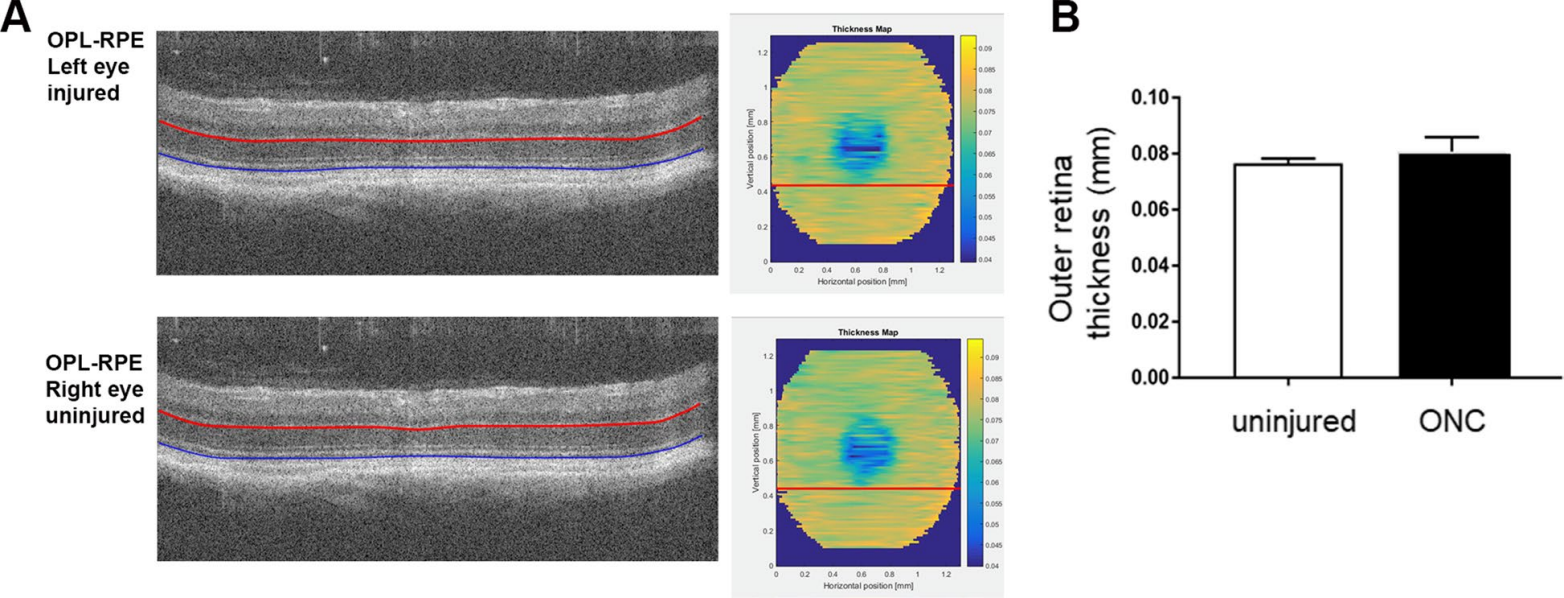
The thickness of the outer nuclear layer is not decreased by optic nerve injury. **A** Representative OCT B scan images. The red line represents the bottom of the OPL and the blue line represents the top of the RPE, which are the parameters used to define and measure the outer retinal thickness. Heat maps generated by segmentation analysis are shown. Colors that approach dark yellow indicate thicker areas of the retina whereas blue indicates thinner areas. The red lines in the heat maps indicate the approximate location in the retina where the representative OCT B scan image was taken from. **B** The thickness of the outer retina in injured and uninjured eyes was quantified by measuring the distance from the OPL to the RPE (inclusive) in eyes with ONC injury and uninjured fellow eyes. There was no difference in outer retina thickness between the injured and uninjured eyes. *n* = 4, *p* = 0.1715, unpaired Student’s t-test

We next measured the effect of optic nerve injury on the structure of the outer retina. The width of the outer retina decreases after photoreceptor damage caused by mutation, light damage, aging and toxin administration [28, 32–34]. Reduced outer nuclear layer thickness is highly correlated with photoreceptor degeneration and decreased photoreceptor function [28]. The thickness of the outer retina in injured and uninjured eyes was quantified by measuring the distance from the OPL to the RPE (inclusive). As shown in Fig. 2, despite the dramatic loss of RGCs and reduced GCL thickness after optic nerve injury, the thickness of the outer retina was not changed. The outer retina thickness in injured eyes was 0.0808 ± 0.0052 mm whereas the uninjured eye was 0.07657 ± 0.0017 mm and the difference between the average thickness for the injured and uninjured eyes was not significant (*n* = 5, *p* = 0.1715, unpaired Student’s t-test). Paired analysis in which intra-ocular differences within each animal were compared also demonstrated that there was no significant difference between eyes (left vs right eyes, *p* = 0.1432, paired t-test, *n* = 5). Therefore, optic nerve injury did not lead to significant structural changes or thinning of the outer retina.

**Fig. 3:**
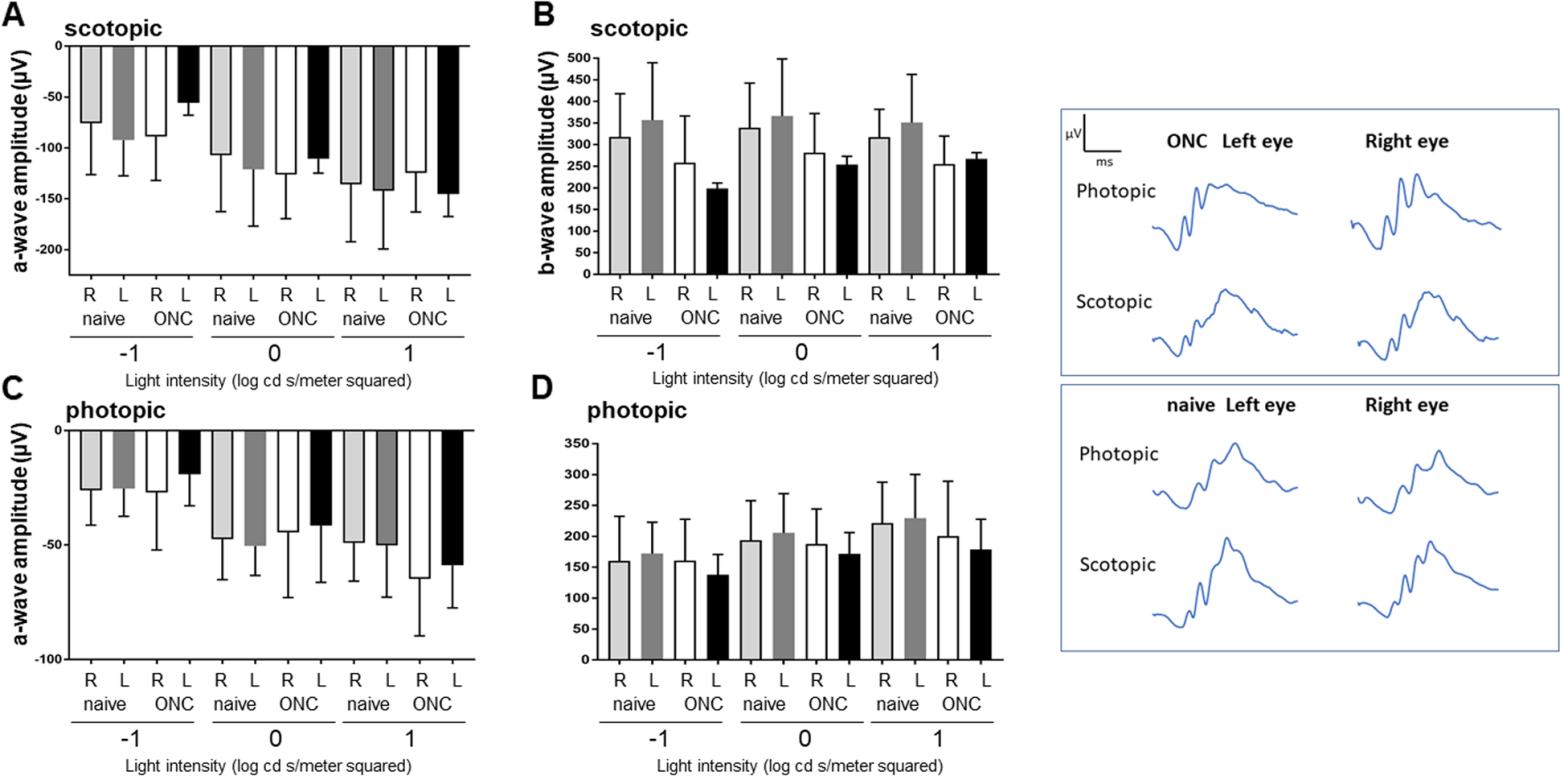
The function of the outer and inner retina is not affected by optic nerve injury. The average responses to three light intensities are shown. Optic nerve crush (ONC) injury did not reduce photoreceptor function at any light intensity. **A** Scotopic a-wave and **B** scotopic b-wave amplitudes were recorded using white light to stimulate rod photoreceptors. The responses from ONC injured left eyes are shown as black bars, uninjured right eyes are shown as white bars, uninjured left eyes from naïve mice are shown as light grey bars, uninjured right eyes from naïve mice are shown as dark grey bars. **C** Photopic a-wave and **D** b-wave amplitudes recorded using flashes of green light to stimulate cone photoreceptors also demonstrate no significant reduction at any light intensity. R, right; L, left; ONC, optic nerve crush. *n* = 3–5, ANOVA with Tukey’s post-hoc. **E** Representative photopic and scotopic ERG waveforms from each treatment group are shown

To determine whether optic nerve injury led to changes in the function of the outer retina, we used flash ERG to measure rod and cone photoreceptor responses (a-waves) and inner retina responses (b-waves, primarily from ON-center bipolar cells [35]). To control for any potential anesthetic effect on ERGs [36] or differences between right and left recording electrodes, we used naïve mice that were anesthetized only without optic nerve injury. The ERG analysis demonstrated that scotopic (rod-driven) and photopic (cone-driven) responses were equivalent between injured optic nerve crush left eyes and uninjured right eyes at all light intensities tested (Fig. 3). For example, the average scotopic a-wave responses at the medium light intensity (1 cd s/m^2^) for injured left eyes was -110.88 μV ± 13.98 and for uninjured right eyes was -126.10 μV ± 43.51; the difference between the eyes was not significant (*p* > 0.99) indicating the injury had no effect on rod photoreceptor function. Similarly, the average photopic a-wave responses in the injured left eyes at 1 cd s/m^2^ light intensity was -41.56 μV ± 24.76, which was not significantly different from the response in the uninjured right eyes, -44.55 μV ± 28.43, indicating no effect of injury on cone photoreceptor responses (*p* > 0.99). Furthermore, there was no significant difference between the average scotopic or photopic a-wave responses at any intensity between the injured left eyes of the experimental optic nerve injury mice and the uninjured left eyes of the naïve animals, again indicating no effect of optic nerve injury on the outer retina. For example, the average scotopic a-wave responses at the medium light intensity (1 cd s/m^2^) for injured left eyes were -110.88 μV ± 13.98 and for uninjured left eyes of the naïve mice was -121.14 μV ± 55.78 (*p* > 0.99), and average photopic a-wave responses at the medium light intensity (1 cd s/m^2^) for injured left eyes was -41.56 μV ± 24.76 and for uninjured left eyes of the naïve mice was -50.43 μV ± 12.84 (*p* > 0.99).

Analysis of the b-wave responses showed similar findings. There were no significant differences between average responses of injured left eyes and uninjured right eyes in the experimental optic nerve crush mice. For example, at the medium scotopic light intensity (1 cd s/m^2^), the average scotopic b-wave amplitude for the injured left eye was 254.03 μV ± 19.06, which was similar to the uninjured right eye, 281.12 μV ± 90.77 (Fig. 3). Similarly, the average photopic b-wave amplitude for the medium light intensity was 171.59 μV ± 34.45 for the injured left eye and 187.72 μV ± 56.62 for the uninjured right eye. Also, there were no significant differences between b-wave amplitudes of left eyes from injured mice and left eyes from uninjured naïve mice. For example, the average scotopic b-wave responses at the medium light intensity (1 cd s/m^2^) for injured left eyes was 254.03 μV ± 19.06 and for uninjured left eyes of the naïve mice was higher but not significantly different at 366.17 μV ± 132.06 (*p* = 0.81), and average photopic b-wave responses at the medium light intensity (1 cd s/m^2^) for injured left eyes was 171.59 μV ± 34.45 and for uninjured left eyes of the naïve mice was 205.52 μV ± 63.66 (*p* > 0.99). Therefore, these data indicate that optic nerve injury did not alter the function of the outer retina.

As expected, there was no difference in the average scotopic a- or b-wave responses or average photopic a- or -b-wave responses between left and right eyes (both uninjured) in the naïve mice at any light intensity (Fig. 3, compare light grey and dark grey bars). Furthermore, there was no difference between the average scotopic or photopic responses for the uninjured right eyes in the experimental mice and the uninjured right eyes in the naïve mice (Fig. 3, compare white and light grey bars).

**Fig. 4:**
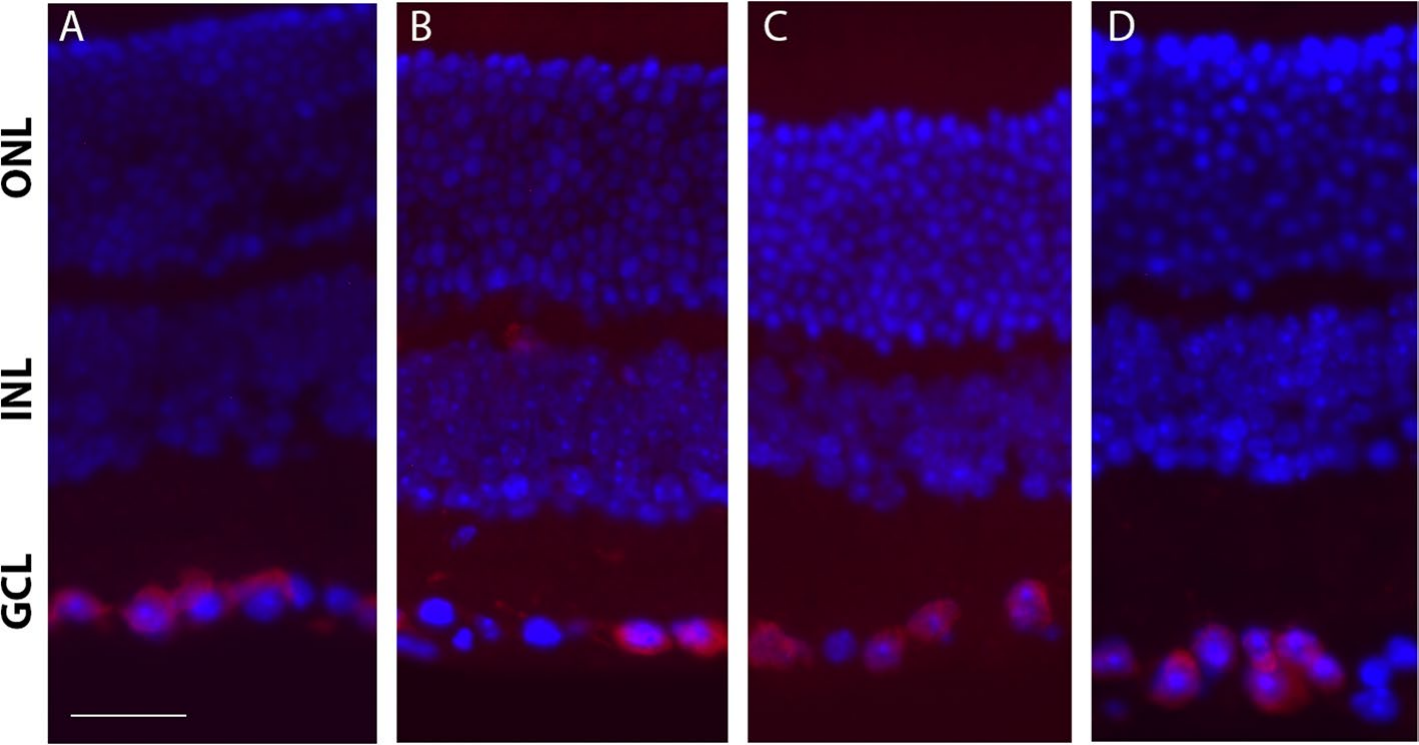
Retinal cross-sections showing normal ONL organization among the different treatments. Immunodetection for the RGC-specific protein RBPMS (red) with nuclei counter-stained using DAPI (blue) shows fewer cells in the GCL in the left eye of injured mice (**B**) compared to uninjured right eyes (**A**). In contrast, normal ONL and INL organization is evident in all treatments. Left eyes (**D**) and right eyes (**C**) of naïve animals are shown for comparison. Scale bar, 50 µm

**Fig. 5:**
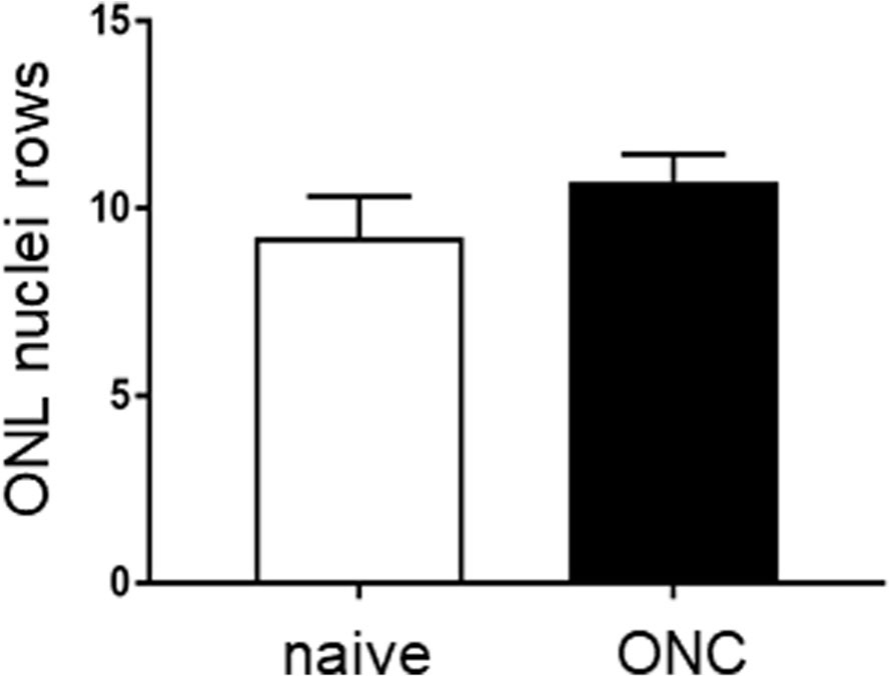
Optic nerve injury did not cause photoreceptor loss. The average number of rows of nuclei in the ONL are shown in ONC (black column) and naïve (white column) eyes. There was no difference in the number of ONL nuclei rows between treatments (*p* = 0.1185, *n* = 3 per group, Student’s unpaired t-test), consistent with the OCT and ERG findings

Additionally, we performed nuclear staining on retinal cross-sections on the injured and uninjured eyes to assess the integrity of the nuclear layers (Fig. 4). There were fewer RBPMS-positive RGCs in the injured eye, as quantified above (Fig. 1C), indicating RGC degeneration and consistent with GCC thinning. In contrast, the ONL of injured eyes and uninjured eyes were similar and showed normal nuclei organization with well-ordered columns. Quantification of ONL nuclei indicated no significant difference in the number of rows between eyes with ONC injured and naïve uninjured eyes (Fig. 5). Therefore, the OCT, ERG and histological analyses indicate that the outer retina was preserved despite substantial RGC loss.

## Discussion

The goal of this study was to determine whether injury to the optic nerve and subsequent axonal atrophy and RGC death leads to detrimental effects on the outer retina, particularly photoreceptor function. This question is important because retaining outer retinal light responses is essential for therapeutic rescue of RGCs after injury. The optic nerve crush model is the most commonly used model for investigating experimental therapies for RGC preservation and axonal regeneration. However, no studies to date have systematically investigated the effect of optic nerve crush injury on the structure and function of the outer retina. Our findings using functional and structural measurements demonstrate that optic nerve injury is limited to the GCL and damage to the outer retina was not detected.

The main conclusion drawn from this study is that the outer retina is preserved and that the injury remains localized and does not spread from the inner to the outer retina. Our finding that the retina appears capable of compartmentalizing injury within the GCL is consistent with previous studies using optic nerve transection or induced glaucoma in other model systems that also showed the outer retina was unaffected [14, 16, 18, 19]. It is possible that the initial site of injury may influence whether other retinal layers are affected. For example, subretinal injection (between RPE and photoreceptor layers) of paraquat, a potent inducer of oxidative stress, caused degeneration of not only the photoreceptor layer but also the INL [28]. This effect of paraquat on the inner retina was likely caused by diffusion of the toxin from the injection site [28]. In contrast, GCL injury in the current study is a secondary effect of optic nerve damage and RGC death does not appear to lead to a bystander effect elsewhere in the retina. Conversely, it is well established that mutations in rod photoreceptor-specific mutations lead to secondary non-autonomous cone degeneration, suggesting that a bystander effect can occur within the outer retina if the initial injury occurs there. This bystander effect has many potential explanations, including reduction of rod-derived viability factor, higher oxygen tension, or non-specific phagocytosis from activated microglia. Future work characterizing the molecular basis of limiting injury to the inner retina after optic nerve crush may ultimately identify potential tissue factors that decrease damage responses. One possibility could involve Muller glia in limiting the spread of injury, perhaps by secreting survival factors that support neurons in both the GCL and ONL or restricting inflammatory responses.

Optic nerve crush injury occurs outside the globe and induces secondary RGC degeneration, which excludes direct effects of the injury on the outer retina. Damage to the vascular supply in the cauterization and optic nerve transection models has been suggested to cause ischemia or glial activation that could also affect the outer retina [21], which would be observed as rapid changes in a-waves. It is also important to note that altered ERG responses often resolve after several days; for example, optic nerve transection and NMDA toxicity to RGCs caused lower a-wave (photoreceptor) amplitudes that returned to baseline by 10 days after injury, although minor reduction in b-wave (bipolar cell) amplitudes were sustained [21]. The two week timepoint analyzed here is typically used with the optic nerve crush injury model and is a week after the maximum rate of RGC loss [1, 2], although it remains possible that photoreceptor changes could occur at later timepoints. Our observation that normal a- and b-waves were detected 2 weeks after optic nerve injury suggests that tissue remodeling and synaptic reorganization that rapidly accompanies many neurodegenerative conditions of the retina [8] did not lead to functional changes in the outer retina. Published studies also indicate that scotopic and photopic ERG responses were normal up to 7 months after optic nerve transection in the rabbit [18], suggesting that the ONL in our model will remain unchanged at later timepoints. Additionally, the numbers of amacrine cells within the GCL did not change when examined up to 15 months after optic nerve transection or ONC [37]. In contrast, reduced photoreceptor function was identified in 2 year old DBA2J mice with late state RGC degeneration [38], although it is unknown whether the photoreceptors were affected directly by the mutated proteins that cause the DBA2J glaucoma-like phenotype, secondary effects of prolonged elevated intraocular pressure, or the exposure of photoreceptors to degenerating RGC and synaptic loss for over a year. Also, cone photoreceptors were lost in the rat laser-photocoagulation-induced ocular hypertension model beginning at 2 weeks after injury and continuing to 6 months [39], consistent with other studies indicating that the specific injury influences the response in the ONL [23]. Future studies will examine whether any delayed effects on photoreceptors are observed several months after ONC.

A survey of the literature indicates species-dependent effects of RGC injury on the ONL. For example, in the present study in the mouse, and reports in rabbit models, flash ERG responses were similar between normal eyes and eyes with optic nerve injuries [18, 19], and multifocal and full field flash ERG responses were only mildly affected in the cat after optic nerve injury [19]. In humans, flash ERGs were normal in eyes with optic nerve lesions [11, 12] and dark-adapted b-wave amplitudes were normal in advanced glaucomatous eyes compared with normal eyes [13] but rod photoreceptor a-waves were lower in advanced glaucoma at high flash intensities [13]. In the macaque, induced glaucoma caused mild but not significant reductions in full field a- and b-wave amplitudes [16]. In contrast, in the rat after optic nerve transection, a-waves were higher and b-waves were reduced in one study [20] and both a- and b-waves were lower in another study [21]. Interestingly, optic nerve transection and intravitreal injections of NMDA in rats protected photoreceptor ERG responses to subsequent light-induced photoreceptor damage [21], similar to findings by [22]. Therefore, the specific RGC injury and the species may influence the effect of RGC death on the ONL.

In conclusion, our analysis did not detect detrimental effects on the outer retina from optic nerve injury despite substantial GCL thinning and RGC degeneration. The findings from this study suggest that investigations of treatments to preserve the GCL with this frequently used optic nerve injury model would not require additional strategies to preserve the ONL. Additionally, these results indicate that tissue reorganization or remodeling in the outer retina as a result of RGC death may be minimal and is likely not an obstacle to therapeutic vision rescue. Our findings also indicate that significant damage does not occur in the fellow uncrushed eye, confirming that the injury response is local to the ON region and GCL of the injured eye and does not induce systemic responses that would affect the function of the uninjured eye. Our results should not be extrapolated to humans but are important for other groups using this injury model and perhaps other injuries targeting the GCL. We encourage all investigators to assess the effect of RGC injuries and experimental therapies on the outer retina as their research progresses to preclinical and clinical trials.

## Data Availability

The datasets used and/or analysed during the current study are available from the corresponding author on reasonable request.

## Abbreviations

ANOVA: Analysis of variance
DAPI: 4′6-Diamidino-2-phenylindole
ERG: Electroretinogram
GCC: Ganglion cell complex
GCL: Ganglion cell layer
INL: Inner nuclear layer
IPL: Inner plexiform layer
OCT: Optical coherence tomography
ONL: Outer nuclear layer
RGC: Retinal ganglion cell
RPE: Retinal pigment epithelium
